# The use of the Labyrinth Scale in the differential diagnosis of children aged 2 to 4 with a history of psychological distress, autism and different language outcomes

**DOI:** 10.1590/2317-1782/e20250152en

**Published:** 2026-05-08

**Authors:** Tainá Rossato Benfica, Fernanda Nunes Franco, Gustavo Marcelino Siquara, Milena Pondé, Ana Paula Ramos de Souza

**Affiliations:** 1 Universidade Federal de Santa Maria – UFSM - Santa Maria (RS), Brasil.; 2 Escola Bahiana de Medicina e Saúde Pública – EBMSP - Salvador (BA), Brasil.; 3 Universidade Federal do Rio Grande do Sul – UFRGS - Porto Alegre (RS), Brasil.

**Keywords:** Autism Spectrum Disorder, Developmental Language Disorder, Differential Diagnosis, Childhood Assessment, Language Delay

## Abstract

**Purpose:**

To analyze the diagnostic utility of the Autism Diagnostic Labyrinth Scale to differentiate children with Autism Spectrum Disorder (ASD) from children with language disorders or delay and those with typical development in the 2 to 4 age range.

**Methods:**

29 children aged 2 to 4 years were evaluated using the Labyrinth Scale, with and without a history of psychological distress, identified as having Typical Development (TD), Delayed Language Acquisition (DLA), Developmental Language Disorder (DLD), or ASD. Totals and subscale scores for social interaction, verbal and nonverbal communication, restricted and repetitive behaviors, and gestures were statistically compared to differentiate between groups.

**Results:**

there were statistically significant differences in total scores and across all four subscales of the Labyrinth Scale between the ASD group and the other clinical groups evaluated (TD, DLA, and DLD) (p < 0.001). The Verbal Communication subscale also demonstrated specific ability to significantly distinguish the LAD group from the DLD group (p<0.001).

**Conclusion:**

The Labyrinth Scale proved to be useful in clearly differentiating children with ASD from those with DLD, LAD, and typical development, suggesting strong clinical potential of the instrument for early differential diagnosis among these clinical conditions.

## INTRODUCTION

The recent publication of the Labyrinth Scale^(1)^ presented it as a new tool to support the diagnosis of Autism Spectrum Disorder (ASD), accessible to health professionals within the national context. The instrument was developed taking into account the clinical practice of experienced professionals in the diagnos

is of ASD, encompassing procedures such as anamnesis and physical and psychological examinations. It uses criteria to establish diagnosis based on the Diagnostic and Statistical Manual (DSM) of the American Psychiatric Association (APA)^(2)^, as well as the International Classification of Diseases (ICD) of the World Health Organization (WHO)^(3)^, which are considered the gold standard for diagnosis.

There are internationally recognized scales, such as the Autism Diagnostic Observation Schedule (ADOS) and the Autism Diagnostic Interview–Revised (ADI-R), whose Portuguese versions already exist but are not available for use, both due to the requirement for specific training and the high cost of the training programs that qualify professionals to administer these scales^(4,5)^. In this context, the Labyrinth Scale emerges as a proposal developed and validated in the Brazilian population, providing greater accessibility for health professionals, among whom speech-language pathologists stand out, as they are often the first to receive very young children with ASD who arrive at the clinic with complaints of delayed language acquisition (DLA). It is also common for these professionals to receive children with diagnoses that raise doubts, especially cases of ASD level I diagnosed as developmental language disorder (DLD ) or, conversely, children with DLD diagnosed as having ASD.

One important aspect in younger children is the difficulty in differentiating ASD from other neurodevelopmental disorders, among which Developmental Language Disorder (DLD) stands out, particularly in cases presenting pragmatic impairments^(6)^. DLD has a multifactorial origin and is characterized, according to the DSM-5, by persistent deficits in the acquisition and use of language, which may affect both receptive and expressive domains^(2,7)^. One of the diagnostic criteria most frequently considered in the literature is delayed language maturation of at least 12 months in relation to the expressive domain in typical language acquisition, and at least six months in the receptive domain^(7)^. Common manifestations include reduced vocabulary, limited sentence structure, difficulties in discourse formulation, and language abilities below expected levels, which create obstacles to the effective use of communication in social contexts and may affect academic performance. Symptoms are present from early development and occur in the absence of sensory or cognitive deficits, global neurodevelopmental delays, or other biological conditions^(2)^. It is estimated that the prevalence of children with language disorders ranges from 3 to 10%, affecting boys more frequently than girls^(8)^.

Another frequent diagnosis in the field of language is DLA, which presents clinical features similar to those of DLD, especially in very young children. However, although the manifestations found in cases of DLD are also present in cases of DLA, the impairments are less persistent and do not characterize deviant processes in relation to typical development; that is, there are no physiopathological signs as observed in cases of DLD^(9)^.

There are also studies^(9,10)^ showing that a history of psychological distress in the first 18 months of life may be associated with outcomes of DLA, in addition to the possibility of detecting early signs of risk for progression to ASD as early as the first year of life^(11)^. A history of psychological distress has also been found in samples of deaf children, highlighting the importance of this perspective in the first year of life^(12)^.

Considering these aspects, this study’s objective is to analyze the diagnostic utility of the Autism Diagnostic Labyrinth Scale in differentiating children with Autism Spectrum Disorder (ASD) from children with language disorder or DLA and those with typical development in the 2 to 4 age range. The study also aims to examine the contributions of the social interaction, verbal and nonverbal communication, restricted behaviors, and repetitive gestures subscales in differentiating the groups.

## METHOD

This is a quantitative, cross-sectional study. The convenience sample consisted of 29 children aged between 2 years and 11 months and 4 years and 11 months, recruited from private clinics, university clinics, and early childhood education schools in medium- and large-sized municipalities in the state of Rio Grande do Sul.

Inclusion criteria comprised children aged between 2 years and 11 months and 4 years and 11 months, with different clinical conditions and a history of language complaints and/or a history of psychological distress assessed using the Clinical Reference Indicators for Child Development – Questionnaire (IRDI-Q)^(13)^. This resulted in four groups: four children with Typical Development (TD) and a history of psychological distress, five children with complaints of Delayed Language Acquisition (DLA) and a history of psychological distress, 10 children with Developmental Language Disorder (DLD), and 11 children with Autism Spectrum Disorder (ASD). Exclusion criteria included the presence of biological alterations such as diagnosed syndromes, malformations, or organic lesions.

### Ethical considerations

This study was submitted to and approved by the Research Ethics Committee (REC) of the Universidade Federal de Santa Maria under protocol number 5,057,051, with CAAE number 52044121.6.0000.5346. All the study’s stages were conducted in accordance with the guidelines and regulatory standards for research involving human beings, as established by the Brazilian National Health Council in Resolutions 466/12 and 510/16. The research participants’ legal guardians read and signed the Free and Informed Consent Term (FICT), agreeing to participate. They also received a confidentiality agreement signed by the principal investigator.

### Instruments and data collection procedures

To assess the history of psychological distress, the IRDI-Q instrument^(13)^ was used. It consists of 31 questions that retrospectively indicate clinical markers of risk to child development and is intended to be applied to children aged between 2 years and 11 months and 7 years. This instrument is a retrospective questionnaire that is independent of the child’s current age or developmental stage, is easy and quick to administer, and has an average administration time of 15 minutes. The tool was developed based on fundamental psychic operations involving the mother–child relationship and is structured around four main axes: Subject Assumption (SA), Demand Establishment (DE), Presence/Absence Alternation (PA), and Paternal Function (PF). Caregivers’ responses to the questionnaire are rated on a scale according to the following options: “never”, “rarely”, “sometimes”, “often”, “always”, or “do not remember”. The cutoff score indicating a history of psychological distress/presence of developmental risk is 31 points or higher. Children scoring 31 points or more who were assessed in municipal schools were invited to undergo evaluation with the Labyrinth Scale^(1)^. Children who attended university clinics or private clinics were also assessed using the IRDI-Q^(13)^ to verify the presence or absence of a history of psychological distress associated with DLD or ASD in the first 18 months of life.

The second instrument used was the Labyrinth Scale^(1)^, which is a diagnostic assessment instrument designed to characterize the clinical signs of ASD and allows for the mapping of other symptoms commonly associated with children who present with the disorder. The scale is subdivided into Core Symptoms, Associated Symptoms, and Associated Somatic Alterations. The instrument enables the analysis of independent and joint play through proposed structured activities, using specific toys and tasks performed by the examiner during interaction with the child. In addition, it includes an anamnesis that details the child’s developmental history with regard to verbal and nonverbal communication, social interaction, family activities and school participation, medical and family history, life history, associated symptoms, and sociodemographic data.

The Core Symptoms’ analysis comprises four axes: Rigid Behavior and Repetitive Gestures, through which symbolic play, rigidity, repetitive movements, and restricted interests are assessed; Nonverbal Communication, composed of the items: response to name being called, eye contact, initiation and response to joint attention, and communicative gestures; Verbal Communication, which includes expressive verbal language items, linguistic repertoire, and reciprocity in communication; and Social Interaction, which includes the items: response to approach, seeking others, and social smiling. Scoring is performed using a Likert Scale ranging from zero to five points. The cutoff score for ASD is 12 points or higher. The critical scores for ASD diagnosis within the domains are: three or more points in social interaction, four or more points in verbal communication, two or more points in nonverbal communication, and four or more points in rigid behavior and repetitive gestures.

Beyond the assessments using the Labyrinth Scale^(1)^ and the IRDI-Q^(13)^, children were differentiated regarding DLA or DLD through specific language tests selected according to the physiopathological signs observed in the clinical analysis conducted by speech-language pathologists. As an initial parameter, the Dimensional Inventory for Child Development Assessment (IDADI)^(14)^ was used. This parent-report instrument allows for a global assessment of neurodevelopmental milestones, including cognitive and socioemotional domains, receptive and expressive communication and language, gross and fine motor skills, as well as adaptive behavior. This assessment identifies whether there is delay in the evaluated domains and the level of severity: alert for delay, delay, or significant delay. In cases of significant delay, speech-language pathologists proceeded with transcription of language interactions, through which they were able to identify physiopathological signs such as anomias, difficulties in syntactic construction or agrammatism, pragmatic inadequacies, or motor signs such as groping and articulatory variability. Based on these signs, they selected and administered more specific assessments of vocabulary, fluency, pragmatics, phonology (ABFW)^(15)^, praxis^(16)^, or more general instruments such as the Language Development Assessment (LDA)^(17)^.

### Data analysis

Descriptive analyses were performed on data from the 29 participants in order to highlight the overall results obtained from both instruments. Considering the diagnostic nature of the Labyrinth Scale, statistical analyses were conducted to compare the four study groups with respect to performance on the subscales and the total score of this scale. Initially, descriptive statistical analyses were carried out for sociodemographic data and scores on the instruments used. To compare instrument scores across the different groups, the nonparametric Kruskal–Wallis test was applied using the Jeffreys’s Amazing Statistics Program (JASP). Identification of differences between each group was calculated using the Conover post hoc test.

## RESULTS

Table 1 presents the main results obtained with the instruments, indicating the children’s condition in terms of medical/speech-language pathology diagnosis, history of psychological distress, and the scores obtained on the Labyrinth Scale, both total and by subscales.

**Table 1:** Description of participating subjects, IRDI-Q and Labyrinth Scale scores, and reported diagnoses

**Subject**	**Age**	**IRDI-Q**	**IRDI-Q status**	**Labyrinth Core Symptoms**					**Labyrinth**		**Professional Diagnosis**
				**SI**	**VC**		**NVC**	**RRB**	**Total**	**Has ASD**	
1	2y11m	24	without distress	12	0	19		13	54	Yes	ASD
2	4y9m	35	with distress	0	0	0		0	0	No	DLA^*^
3	4y2m	33	with distress	2	3	2		0	7	No	DLA
4	3y6m	39	with distress	0	0	2		0	2	No	TD
5	4y2m	43	with distress	3	1	1		0	5	No	TD
6	4y1m	59	with distress	2	0	0		1	3	No	TD
7	4y6m	35	with distress	0	0	0		0	0	No	TD
8	4y4m	40	with distress	2	3	2		1	8	No	TD
9	4y7m	43	with distress	0	2	0		0	2	No	DLA
10	3y5m	30	without distress	0	4	5		0	9	No	DLD
11	3y	34	with distress	1	8	5		1	15	No	DLD
12	2y11m	33	with distress	0	3	1		0	4	No	DLD
13	2y11m	19	without distress	1	7	7		1	16	No	DLD
14	2y11m	60	with distress	3	9	5		3	20	Yes	ASD
15	3y3m	46	with distress	11	0	22		11	54	Yes	ASD
16	4y9m	40	with distress	3	5	2		3	13	No	DLD
17	3y11m	32	with distress	0	7	1		0	8	No	DLD
18	3y10m	49	with distress	0	0	1		0	1	No	DLA*
19	3y10m	44	with distress	0	5	3		0	8	No	DLD
20	3y10m	41	with distress	2	8	6		2	18	No	DLD
21	3y2m	33	with distress	5	9	13		5	32	Yes	ASD
22	3y	71	with distress	9	9	14		9	38	Yes	ASD
23	4y4m	57	with distress	5	9	7		5	26	Yes	ASD
24	4y11m	18	without distress	0	9	0		0	9	No	DLD
25	3y4m	27	without distress	2	9	3		2	16	No	DLD
26	3y6m	56	with distress	4	7	14		4	29	Yes	ASD
27	4y6m	52	with distress	1	8	5		1	15	No	DLA
28	3y	23	without distress	2	5	5		2	14	No	ASD
29	4y	59	with distress	6	1	11		6	34	Yes	ASD

* Had DLA but no longer has it

Caption: DLA = Delayed Language Acquisition; DLD = Developmental Language Disorder; TD = Typical Development; ASD = Autism Spectrum Disorder; SI = Social Interaction; VC = Verbal Communication; NVC = onverbal Communication; RRB = Rigid and Repetitive Behaviors

In the studied sample (Table 1), of the 29 participants evaluated, 9 children (32%) received a medical diagnosis of Autism Spectrum Disorder (ASD), 10 children (36%) were diagnosed with Developmental Language Disorder (DLD), 5 children (18%) presented Typical Development (TD), and 4 children (14%) were diagnosed with Delayed Language Acquisition (DLA).

Of the 9 children with a medical diagnosis of ASD, 8 (88.9%) met the diagnostic criteria on the Labyrinth Scale. Only one child (11.1%) did not meet the diagnostic criterion on this scale in the Social Interaction item (Subject 28). According to the therapist, this child has ASD level 1 and has received intervention since 11 months of age, which may explain the score below the cutoff point.

**Figure 1:**
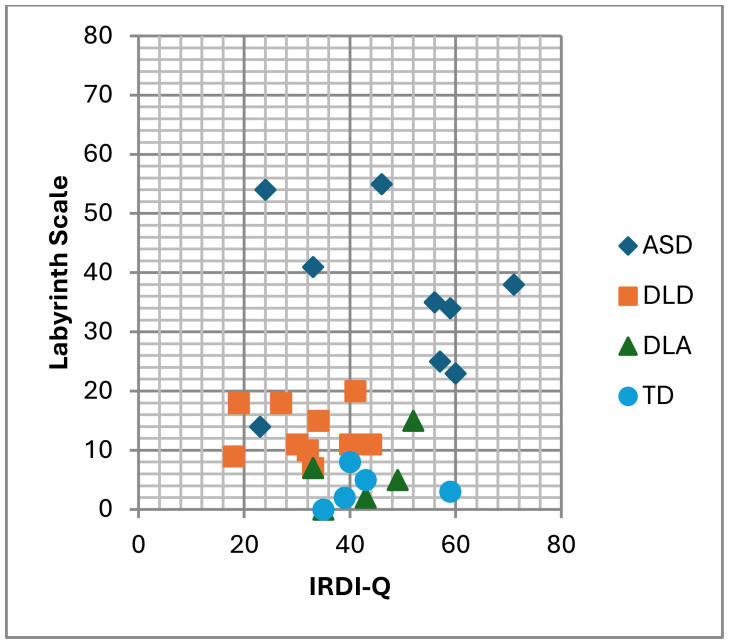
Distribution of subjects in assessments based on diagnosis

Figure 1 shows the distribution of subjects, indicating that children diagnosed with ASD presented scores above the cutoff point of 12 points on the Labyrinth Scale (8 children, 88.9%) and predominantly scores above 33 points on the IRDI-Q (7 children, 77.8%). Among subjects with TD (n=5), DLA (n=4), and DLD (n=10), despite having a history of psychological distress (scores between 33 and 60 on the IRDI-Q), development did not indicate severe psychopathology at the time of assessment.

Caption: DLA = Delayed Language Acquisition; TD = Typical Development; DLD = Developmental Language Disorder; ASD = Autism Spectrum Disorder

Regarding scores on the Labyrinth Scale, the lowest mean scores were obtained by children with TD (Mean=3.6; SD=3.0), followed by the DLA group (Mean=5.8; SD=5.8) and the DLD group (Mean=12.7; SD=4.5). The highest scores were found in the ASD group (Mean=35.4; SD=13.6), as shown in Table 2.

**Table 2:** Means and standard deviations of the clinical groups on the Labyrinth Scale

**Clinical Groups**	**TD**		**DLA**		**DLD**		**ASD**		**Kruskal-Wallis P value**
	**M**	**SD**	**M**	**SD**	**M**	**SD**	**M**	**SD**	
Social Interaction	1.4	1.3	0.6	0.8	0.9	1.1	6.3	3.5	< .001
Verbal Communication	0.8	1.3	2.6	3.2	6.5	2.1	8.7	1.7	< .001
Nonverbal Communication	1.0	1.0	1.6	2.0	3.3	2.3	12.2	5.9	< .001
Rigid and Repetitive Behaviors	0.4	0.5	0.2	0.4	0.9	1.1	6.4	3.7	< .001
Labyrinth Scale	3.6	3.0	5.8	5.8	12.7	4.5	35.4	13.6	

Caption: TD = Typical Development; DLA = Delayed Language Acquisition; DLD = Developmental Language Disorder; ASD = Autism Spectrum Disorder; M = Mean; SD = Standard Deviation

In Table 3, statistical comparisons were performed between the clinical groups (Kruskal–Wallis test followed by Conover’s post hoc test).

**Table 3:** Comparison using the Conover post hoc test between pairs of clinical groups across the subscales and total score of the Labyrinth Scale

**Labyrinth Scale Subscales and Total**	**Group Comparison**	**z**	**W _i_**	**W _j_**	**p**
Verbal Communication	TD - DLA	-0.63	480	8.20	0.268
	TD - DLD	-2.48	4.80	16.30	0.262
	TD - ASD	-3.86	4.80	23.00	0.006^*^
	DLA - DLD	-1.75	8.20	16.30	< .001^**^
	DLA - ASD	-3.14	8.20	23.00	0.040*
	DLD - ASD	-1.72	16.30	23.00	< .001**
Social Interaction	TD - DLA	0.76	13.00	9.00	0.223
	TD - DLD	0.53	13.00	10.55	0.295
	TD - ASD	-2.46	13.00	24.38	0.007*
	DLA - DLD	-0.34	9.00	10.55	0.366
	DLA - ASD	-3.32	9.00	24.38	< .001**
	DLD - ASD	-3.6	10.55	24.38	< .001**
Nonverbal Communication	TD - DLA	-0.24	7.30	8.60	0.404
	TD - DLD	-1.40	7.30	13.80	0.080
	TD - ASD	-3.57	7.30	24.16	< .001*
	DLA - DLD	-1.12	8.60	13.80	0.131
	DLA - ASD	-3.30	8.60	24.16	< .001**
	DLD - ASD	-2.67	13.80	24.16	0.0004**
Rigid and Repetitive Behaviors	TD - DLA	0.32	9.90	8.20	0.371
	TD - DLD	-0.51	9.90	12.20	0.304
	TD - ASD	-3.24	9.90	24.72	< .001**
	DLA - DLD	-0.89	8.20	12.20	0.186
	DLA - ASD	-3.62	8.20	24.72	< .001**
	DLD - ASD	-3.33	12.20	24.72	< .001**
Labyrinth Scale total score	TD - DLA	-0.42	5.30	7.60	0.335
	TD - DLD	-2.08	5.30	15.00	0.019*
	TD - ASD	-4.04	5.30	24.50	< .001**
	DLA - DLD	-1.58	7.60	15.00	0.056
	DLA - ASD	-3.56	7.60	24.50	< .001**
	DLD - ASD	-2.43	15.00	24.50	0.008*

* p<0.05;

** p<0.01

Caption: TD = Typical Development; DLA = Delayed Language Acquisition; DLD = Developmental Language Disorder; ASD = Autism Spectrum Disorder; W_i_ and W_j_ = sum of items score; z = number of standard deviations above (positive) or below (negative)

The results in Table 3 show that, in the Verbal Communication subscale, there were significant differences between the TD-ASD (p=0.006), DLA–DLD (p<0.001), DLA-ASD (p=0.040), and DLD-ASD (p<0.001) groups. For the Social Interaction subscale, significant differences were observed between TD-ASD (p=0.007), DLA-ASD (p<0.001), and DLD-ASD (p<0.001). In the Nonverbal Communication subscale, significant differences were found between TD-ASD (p<0.001), DLA-ASD (p<0.001), and DLD-ASD (p=0.0004). In the Restricted Behaviors and Repetitive Gestures subscale, significant differences were observed between TD-ASD (p<0.001), DLA-ASD (p<0.001), and DLD-ASD (p<0.001).

In the Total Score analysis of the Labyrinth Scale, there were significant differences between TD-DLD (p=0.019), TD-ASD (p<0.001), DLA-ASD (p<0.001), and DLD-ASD (p=0.008).

**Figure 2:**
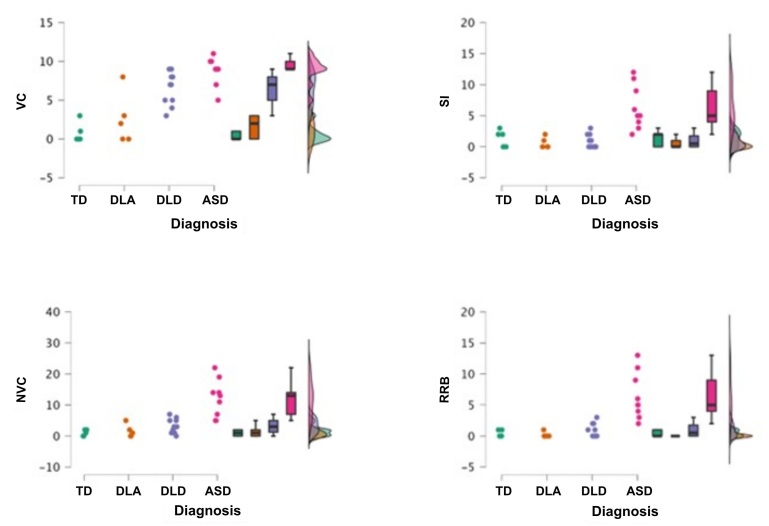
Comparison of total subscale scores

**Figure 3:**
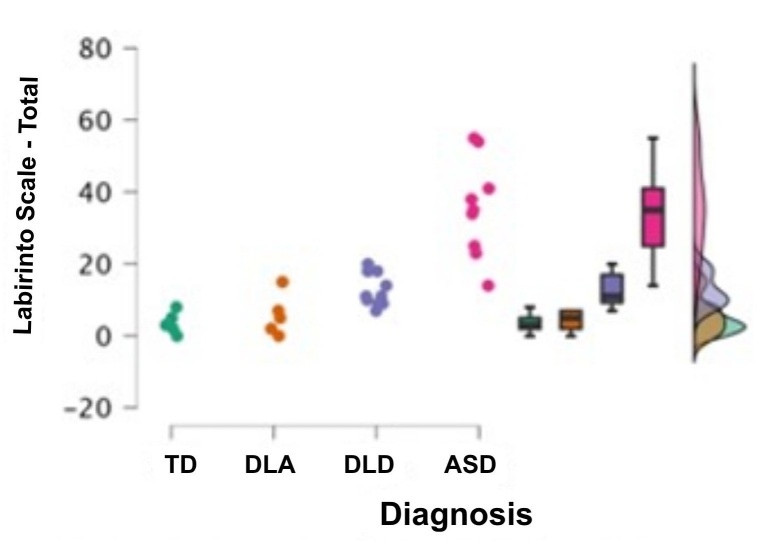
Comparison of groups in total Labyrinth Scale scores

These results are visually represented in Figures 2 and 3, which clearly show the progressive increase in the total Labyrinth Scale score from the group with typical development to the group diagnosed with ASD.

Caption: VC = Verbal Communication; SI = Social Interaction; NVC = Nonverbal Communication; RRB = Rigid and Repetitive Behaviors; TD = Typical Development; DLA = Delayed Language Acquisition; DLD = Developmental Language Disorder; ASD = Autism Spectrum Disorder

Caption: TD = Typical Development; DLA = Delayed Language Acquisition; DLD = Developmental Language Disorder; ASD = Autism Spectrum Disorder

Figure 2 visually presents the comparison among the four clinical groups (TD, DLA, DLD, and ASD) for each subscale assessed by the Labyrinth Scale (Verbal Communication, Social Interaction, Nonverbal Communication, Rigid Behavior and Repetitive Gestures). It is clearly observed that the group diagnosed with ASD presents higher scores across all subscales compared to the other three groups.

More specifically:

- Verbal Communication: Children with ASD showed a significantly higher mean (Mean=8.7; SD=1.7), followed by children with DLD (Mean=6.5; SD=2.1), DLA (Mean=2.6; SD=3.2), and TD (Mean=0.8; SD=1.3).
- Social Interaction: Children with ASD obtained higher scores (Mean=6.3; SD=3.5), standing out in relation to children with DLD (Mean=0.9; SD=1.1), TD (Mean=1.4; SD=1.3), and DLA (Mean=0.6; SD=0.8).
- Nonverbal Communication: The ASD group showed significantly higher means (Mean=12.2; SD=5.9) compared to the DLD (Mean=3.3; SD=2.3), DLA (Mean=1.6; SD=2.0), and TD (Mean=1.0; SD=1.0) groups.
- Rigid Behavior and Repetitive Gestures: Again, the ASD group showed the highest scores (Mean=6.4; SD=3.7), followed by the DLD (Mean=0.9; SD=1.1), TD (Mean=0.4; SD=0.5), and DLA (Mean=0.2; SD=0.4) groups.

These data visually reinforce the discriminative potential of the Labyrinth Scale subscales, highlighting the distinct profile of children with ASD in comparison with the other groups analyzed.

Figure 3 visually presents the comparison among the total scores of the clinical groups (TD, DLA, DLD, and ASD) obtained on the Labyrinth Scale. A clear progressive increase in total Labyrinth Scale scores across the clinical groups can be observed.

More specifically:

- The Typical Development (TD) group obtained the lowest mean total score (Mean=3.6; SD=3.0), followed by the Delayed Language Acquisition (DLA) group (Mean=5.8; SD=5.8), the Developmental Language Disorder (DLD) group (Mean=12.7; SD=4.5), culminating in the group diagnosed with ASD, which presented the highest mean total score (Mean=35.4; SD=13.6).

This visualization reinforces the descriptive finding obtained through the statistical analysis (Table 3), which showed particularly clear significant differences between the ASD group and the other clinical groups evaluated (TD, DLA, DLD).

## DISCUSSION

The study’s results demonstrated that the Labyrinth Scale^(1)^ was able to clearly differentiate children diagnosed with Autism Spectrum Disorder (ASD) from the other clinical conditions evaluated (DLD, DLA, and TD). The findings indicated significant differences in the Verbal Communication subscale between children with ASD and the other groups, as well as between the groups of children with Delayed Language Acquisition (DLA) and Developmental Language Disorder (DLD). Furthermore, the Social Interaction (SI), Nonverbal Communication (NVC), and Rigid and Repetitive Behaviors (RRB) subscales stood out for their ability to discriminate children with ASD from the other groups. A significant history of psychological distress was also observed on the IRDI-Q instrument^(13)^ across all groups, being more pronounced among children with ASD.

The findings highlight that the Verbal Communication subscale of the Labyrinth Scale is sensitive in the clinical differentiation between children with ASD and those with DLD, DLA, and TD. In particular, a distinction was also noted between children with DLA and DLD, indicating this subscale as useful for the specific clinical differentiation between groups with language complaints. These findings corroborate the results reported by Hage et al.^(6)^, who also identified substantial differences in pragmatic abilities among children with ASD, DLD, and TD, indicating greater pragmatic impairment in children with ASD due to their more pronounced communicative and social difficulties^(6)^. The findings of the present study reinforce the clinical importance of a detailed assessment of verbal abilities, contributing to early identification and differential diagnosis of ASD in relation to isolated language difficulties, especially in young children.

The SI, NVC, and RRB subscales revealed strong discriminative potential for the ASD group compared to the other clinical groups (DLD, DLA, and TD). Only one child (subject 28) did not meet diagnostic criteria, which may be associated with the early intervention received. These findings are consistent with previous studies that validated similar instruments, such as the ADOS and the ADI-R, which emphasize the relevance of social, communicative, and behavioral deficits for the differential diagnosis of ASD^(4,5,12)^. This study reinforces the Labyrinth Scale value as a practical and effective instrument for the early recognition of typical ASD characteristics, as it is an accessible, easy-to-administer, and low-cost tool, validated specifically for the Brazilian clinical context^(1)^.

This study also identified that children with a history of psychological distress (assessed using the IRDI-Q)^(13)^ may develop without severe clinical manifestations, later presenting typical development or only mild language difficulties. These results are consistent with the findings of Kupfer et al.^(18)^, Nunes et al.^(10)^, and other subsequent studies^(19-21)^, which emphasize that the early presence of psychological distress does not always indicate a pathological developmental trajectory. Nevertheless, the presence of such distress requires specialized clinical attention, with early and interdisciplinary interventions that may promote more favorable outcomes^(22)^. Thus, these data highlight the importance of early identification and integrated intervention with a focus on the family and the promotion of child mental health, directly informing preventive clinical practices in primary health care.

Another relevant aspect observed in this study concerns the coexistence of psychological distress with language delays and disorders (DLA and DLD). Souza et al.^(9)^ also identified associations between early psychological distress and language delays, suggesting a unique connection between psychological and linguistic development. Santos et al.^(23)^ reinforce that the intersubjective dimension significantly influences language acquisition, even though a direct causal relationship with neurodevelopmental disorders is not established. The results presented here reiterate that linguistic and psychological development should be assessed jointly, promoting an interdisciplinary clinical practice that is sensitive to different trajectories of child development^(22,23)^.

Considering the limitations of this study, particularly the small number of participants due to the pandemic context, future research with larger samples is recommended in order to deepen the analysis of the differential diagnostic contribution of the Labyrinth Scale between ASD and DLD. In addition, broader population-based studies on psychological and linguistic aspects in the differentiation between DLA and DLD are suggested, contributing to a more refined understanding of environmental and family factors that influence the emergence and progression of these clinical conditions.

This study contributes to Brazilian interdisciplinary child clinical practice, with direct implications for primary and secondary health care. Instruments such as the IRDI-Q^(13)^ proved effective for identifying a history of psychological distress, and the Labyrinth Scale^(1)^ proved useful in the differential diagnosis among neurodevelopmental disorders that are often confused in clinical practice. The fact that the Labyrinth Scale is easily accessible to any childhood therapist, has an interdisciplinary character, is low cost, and is validated for the Brazilian population allows us to affirm its potential clinical utility both in differential diagnosis and in monitoring the progression of clinical cases.

With specific regard to language, the verbal communication item of the Labyrinth Scale proved to be productive both in differentiating delayed language acquisition from developmental language disorder and in differentiating these two conditions from ASD. Fluency in dialogue, the presence of stereotyped language, and the linguistic repertoire considered based on developmental language milestones between 2 and 4 years of age allow differentiation among the three conditions, as children with DLA and DLD differ from each other in terms of developmental milestones, with children with DLD presenting more robust alterations and much higher mean scores on this item. They also show verbal communication that differs from that of children with ASD across the three items, particularly with regard to dialogue and the presence of stereotypies. Higher mean scores indicate greater alteration in the subscale.

Another results’ potentially interesting aspect was the distinction observed in nonverbal communication means, in which the means of children with typical development were similar to those of children with DLA. In contrast, children with DLD presented means that were considerably higher than those groups and substantially lower than those of children with ASD. It is known that studies on the contribution of gesture to language acquisition reinforce a multimodal perspective of language^(24)^. This finding demonstrates that nonverbal and verbal communication are integrated within a broader framework of language functioning and that the distinctions found in this sample signal a potential differentiation between children with DLA and DLD in the domain of gestural communication. This result opens perspectives for future research.

## CONCLUSION

Considering the initial study’s objective, it is concluded that the Labyrinth Scale proved to be effective in differentiating the investigated groups (ASD, DLD, DLA, and TD), both in terms of total score and the specific subscales assessed. The results indicate that this instrument has potential clinical utility, particularly in the differential diagnosis between Autism Spectrum Disorder (ASD) and Developmental Language Disorder (DLD). Furthermore, the Verbal Communication subscale demonstrated specific ability to distinguish children with ASD, DLD, and DLA, suggesting that its complementary use alongside specific language tests may contribute to a more precise and refined assessment, assisting speech-language pathologists in the clinical differentiation of these conditions in practice.
