# Supplementary material for: The use of the Labyrinth Scale in the differential diagnosis of children aged 2 to 4 with a history of psychological distress, autism and different language outcomes
Source: Codas. 2026 May 8;38(2):e20250152. doi: 10.1590/2317-1782/e20250152en (PMC13192452; doi:10.1590/2317-1782/e20250152en)
Supplement: Translation

# Translation: A utilização da Escala Labirinto no diagnóstico diferencial de crianças de 2 a 4 anos com histórico de sofrimento psíquico, autismo e distintos desfechos de linguagem

**DOI:** 10.1590/2317-1782/e20250152pt

**Authors:** Tainá Rossato Benfica, Fernanda Nunes Franco, Gustavo Marcelino Siquara, Milena Pondé, Ana Paula Ramos de Souza

**Affiliations:** 1 Universidade Federal de Santa Maria – UFSM - Santa Maria (RS), Brasil.; 2 Escola Bahiana de Medicina e Saúde Pública – EBMSP - Salvador (BA), Brasil.; 3 Universidade Federal do Rio Grande do Sul – UFRGS - Porto Alegre (RS), Brasil.

**Keywords:** Transtorno do Espectro Autista, Transtorno do Desenvolvimento da Linguagem, Diagnóstico Diferencial, Avaliação na Infância, Atraso de Linguagem

## Abstract

**Objetivo:**

Analisar a utilidade diagnóstica da Escala Labirinto de Diagnóstico de Autismo para diferenciar crianças com Transtorno do Espectro do Autismo (TEA) de crianças com transtorno ou atraso de linguagem e desenvolvimento típico na faixa etária de 2 a 4 anos.

**Método:**

Foram avaliadas 29 crianças de 2 a 4 anos utilizando-se a Escala Labirinto abrangendo casos com e sem histórico de sofrimento psíquico, identificadas com desenvolvimento típico (DT), atraso na aquisição da linguagem (AL), transtorno do desenvolvimento da linguagem (TDL) ou TEA. As pontuações totais e nas subescalas de interação social, comunicação verbal e não verbal, comportamentos e gestos restritos e repetitivos foram comparadas estatisticamente na diferenciação dos grupos.

**Resultados:**

Houve diferenças estatisticamente significativas nas pontuações totais e em todas as quatro subescalas da Escala Labirinto entre o grupo com TEA e os demais grupos clínicos avaliados (DT, AL e TDL) (p < 0,001). A subescala Comunicação Verbal também apresentou capacidade específica para distinguir significativamente o grupo com AL do grupo com TDL (p < 0,001).

**Conclusão:**

A Escala Labirinto demonstrou-se útil para diferenciar claramente crianças com TEA das crianças com TDL, AL e desenvolvimento típico, sugerindo forte potencial clínico do instrumento no diagnóstico diferencial precoce entre esses quadros clínicos.

## INTRODUÇÃO

A recente publicação da Escala Labirinto^(1)^ apresentou-a como uma nova ferramenta para o auxílio ao diagnóstico do Transtorno do Espectro do Autismo (TEA) acessível aos profissionais da saúde na realidade nacional. O instrumento foi construído levando em conta a prática de clínicos experientes no diagnóstico de TEA, abrangendo procedimentos como anamnese, exames físico e psíquico. Ela utiliza critérios para estabelecer o diagnóstico a partir do Manual Diagnóstico e Estatístico da Associação Americana de Psiquiatria (DSM)^(2)^, assim como a Classificação Internacional de Doenças (CID) da Organização Mundial da Saúde (OMS)^(3)^, considerados padrão ouro para o diagnóstico.

Existem escalas mundialmente reconhecidas, como a *Autism Diagnostic Observation Schedule* (ADOS) e *Autism Diagnostic Interview-Revised* (ADI-R), cujas versões em português já existem, mas não estão disponíveis para uso, tanto devido à demanda por formação específica, quanto pelo elevado custo para realizar as formações que habilitam o profissional a aplicar tais escalas^(4,5)^. A partir de tal realidade, a Escala Labirinto desponta como uma proposta criada e validada na população brasileira que fornece maior acessibilidade para os profissionais da saúde, entre os quais se destacam os fonoaudiólogos que, muitas vezes, são os primeiros a receberem crianças muito pequenas com TEA e que chegam à clínica com queixa de atraso na aquisição da linguagem. Também é comum receberem crianças com diagnósticos que geram dúvidas, sobretudo de casos de TEA nível de suporte I diagnosticados como TDL ou o contrário, crianças com TDL diagnosticadas como sendo TEA.

Um dos aspectos importantes, nas crianças mais novas é a dificuldade em diferenciar o TEA de outros transtornos do neurodesenvolvimento, entre os quais o Transtorno do Desenvolvimento da Linguagem (TDL), sobretudo os que apresentam comprometimentos pragmáticos^(6)^. O TDL tem origem multifatorial e é caracterizado, segundo DSM-V, pelos déficits persistentes na aquisição e uso da linguagem, podendo acometer tanto o domínio receptivo, quanto o expressivo^(2,7)^. Um dos critérios diagnósticos mais considerado na literatura é a maturação da linguagem atrasada em ao menos 12 meses em relação ao domínio expressivo na aquisição típica de linguagem, e, em no mínimo seis meses, no domínio receptivo^(7)^. Dentre as manifestações comumente encontradas estão vocabulário reduzido, limitação na estrutura das frases, dificuldades de formulação do discurso, capacidades linguísticas abaixo do esperado, que geram impasses no uso efetivo da comunicação no contexto social e podem afetar o desempenho acadêmico. Os sintomas se apresentam desde o início do desenvolvimento e ocorrem na ausência de déficits sensoriais ou cognitivos, atrasos globais de neurodesenvolvimento ou outras condições biológicas^(2)^. Estima-se que a prevalência de crianças que apresentam transtornos de linguagem varia de 3 a 10%, acometendo mais meninos do que meninas^(8)^.

Outro diagnóstico frequente no âmbito da linguagem, é o atraso na aquisição da linguagem, que se apresenta em quadros clínicos de modo semelhante ao TDL, principalmente em crianças muito pequenas. Porém, apesar das manifestações encontradas nos casos de TDL se fazerem presentes nos casos de atraso na aquisição da linguagem, os prejuízos são menos persistentes e não caracterizam processos desviantes dos esperados no desenvolvimento típico, ou seja, não há sinais fisiopatológicos como nos casos de TDL^(9)^.

Há, também, estudos^(9,10)^ que evidenciam que o histórico de sofrimento psíquico nos primeiros 18 meses de vida pode estar associado a desfechos de atraso na aquisição da linguagem, além da possibilidade de se detectar sinais precoces de risco para evolução para um quadro de TEA já no primeiro ano de vida^(11)^. O histórico de sofrimento psíquico também foi encontrado em amostra de crianças surdas, demonstrando a importância deste olhar no primeiro ano de vida^(12)^.

Considerando tais aspectos, o objetivo deste estudo é analisar a utilidade diagnóstica da Escala Labirinto de Diagnóstico de Autismo para diferenciar crianças com Transtorno do Espectro do Autismo (TEA) de crianças com transtorno ou atraso de linguagem e desenvolvimento típico na faixa etária de 2 a 4 anos. Também se objetiva observar as contribuições das subescalas de interação social, comunicação verbal e não verbal, comportamentos restritos e gestos repetitivos na diferenciação dos grupos.

## MÉTODO

Esta pesquisa é quantitativa e transversal. A amostra de conveniência foi constituída de 29 crianças na faixa etária de dois anos e 11 meses a quatro anos e 11 meses, provenientes de clínicas privadas, clínicas-escola e escolas de ensino infantil, dos municípios de grande e médio porte do Rio Grande do Sul.

Os critérios de inclusão foram crianças na faixa etária de dois anos e onze meses a quatro anos e 11 meses, com diferentes condições clínicas com histórico de queixa de linguagem e/ou histórico de sofrimento psíquico avaliado pelos Indicadores Clínicos de Referência ao Desenvolvimento Infantil – Questionário (IRDI-Q)^(13)^. Disso resultaram quatro grupos: quatro crianças com desenvolvimento típico e histórico de sofrimento psíquico (DT), cinco crianças com queixas de atraso na aquisição da linguagem (AL) e histórico de sofrimento psíquico, 10 crianças com Transtorno do Desenvolvimento da Linguagem (TDL) e 11 crianças com Transtorno do Espectro do Autismo (TEA). Os critérios de exclusão foram não ter alterações biológicas como síndromes, malformações ou lesões orgânicas diagnosticadas.

### Considerações éticas

Este projeto foi submetido à aprovação no Comitê de Ética em Pesquisa da Universidade Federal de Santa Maria e aprovado sob o número de protocolo 5.057.051 com número de CAAE 52044121.6.0000.5346. As etapas do estudo foram realizadas conforme as diretrizes e normas regulamentadoras de pesquisas envolvendo seres humanos, no que determina o Conselho Nacional de Saúde em suas resoluções 466/12 e 510/16. Os responsáveis pelos participantes da pesquisa leram e assinaram o Termo de Consentimento Livre e Esclarecido (TCLE), aceitando participar. Receberam o termo de confidencialidade assinado pelo pesquisador responsável.

### Instrumentos e procedimentos de coleta de dados

Para avaliar o histórico de sofrimento psíquico foi utilizado o instrumento IRDI-Q^(13)^, composto por 31 perguntas que sinalizam, retrospectivamente, marcadores clínicos de risco ao desenvolvimento infantil, previsto para ser aplicado em crianças na faixa etária de dois anos e 11 meses a sete anos. Este instrumento é um questionário retrospectivo que independe da idade atual das crianças ou da etapa do desenvolvimento, de fácil e rápida aplicação, com tempo médio de 15 minutos. A ferramenta foi construída embasada nas operações psíquicas fundamentais, que envolvem a relação mãe-filho e está estruturada em quatro eixos principais: suposição de um sujeito (SS), estabelecimento da demanda (ED), alternância presença/ausência (PA), e função paterna (FP). As respostas dos responsáveis ao questionário são classificadas em escala, conforme as opções: “nunca”, “raramente”, “às vezes”, “muitas vezes”, “sempre” ou “não lembro”. O ponto de corte que sinaliza histórico de sofrimento psíquico /presença de risco ao desenvolvimento é de 31 pontos ou mais. As crianças com 31 ou mais pontos avaliadas nas escolas municipais foram chamadas para a avaliação com a Escala Labirinto^(1)^. As crianças que chegaram às clínicas-escola ou privadas foram avaliadas também pelo IRDI-Q^(13)^ para verificar a presença ou não de histórico de sofrimento psíquico associado ao TDL ou TEA nos primeiros 18 meses de vida.

O segundo instrumento utilizado foi a Escala Labirinto^(1)^, que consiste em um instrumento de avaliação diagnóstica e de caracterização dos sinais clínicos de TEA, que permite o mapeamento de outros sintomas que estão comumente associados a crianças que apresentam o transtorno. A escala é subdividida em Sintomas Centrais, Sintomas Associados e Alterações Somáticas Associadas. O instrumento permite a análise do brincar independente e conjunto, por meio de proposta de atividades estruturadas, com uso de determinados brinquedos e tarefas, realizadas pelo avaliador durante interação com a criança. Além disso, contém anamnese que detalha o histórico do desenvolvimento infantil quanto à comunicação verbal e não verbal, interação social, atividades familiares e inserção na escola, antecedentes médicos e familiares, história de vida, sintomas associados e dados sociodemográficos.

Na análise dos Sintomas Centrais encontram-se quatro eixos: Comportamento Rígido e Gesto Repetitivo, por meio dos quais são avaliados o brincar simbólico, rigidez, movimentos repetitivos e interesses restritos; Comunicação Não Verbal, composta pelos itens: resposta ao chamado do nome, contato visual, intenção e resposta da atenção compartilhada e gestos comunicativos; Comunicação Verbal, que contempla itens de linguagem verbal expressiva, repertório linguístico e reciprocidade na comunicação; Interação Social, que contém os itens: resposta a aproximação, busca do outro e sorriso social. A pontuação é realizada pela Escala Likert, entre zero e cinco pontos. O ponto de corte para TEA é de 12 pontos ou mais. A pontuação crítica para diagnóstico de TEA nos domínios são: três ou mais pontos em interação social, quatro ou mais pontos em comunicação verbal, dois ou mais pontos em comunicação não verbal e quatro ou mais pontos em comportamento rígido e gesto repetitivo.

Além das avaliações com a Escala Labirinto^(1)^ e IRDI-Q^(13)^, as crianças foram diferenciadas em relação a terem atraso na aquisição da linguagem ou TDL por meio de testes específicos de linguagem escolhidos de acordo com os sinais fisiopatológicos observados na análise clínica das fonoaudiólogas. Como parâmetro inicial, utilizou-se o Inventário Dimensional de Avaliação do Desenvolvimento Infantil (IDADI)^(14)^, que é respondido pelos pais, a partir do qual se pode avaliar globalmente marcos do neurodesenvolvimento que incluem os domínios cognitivo, socioemocional, a comunicação e a linguagem receptiva e expressiva, a motricidade ampla e fina, bem como o comportamento adaptativo. Essa avaliação identifica se há ou não atraso nos domínios avaliados e o nível de gravidade: se há alerta para atraso, atraso ou atraso significativo. Diante do atraso significativo, os fonoaudiólogos procederam a uma transcrição das interações de linguagem por meio das quais puderem identificar sinais fisiopatológicos como anomias, dificuldades na construção sintática ou agramatismos, inadequações pragmáticas, ou sinais motores como tateio e variabilidade articulatória. A partir desses sinais puderam escolher e aplicar avaliações mais específicas de vocabulário, fluência, pragmática, fonologia (ABFW)^(15)^, praxias^(16)^, ou gerais como a avaliação do desenvolvimento da linguagem (ADL)^(17)^.

### Análise de dados

Foram realizadas análises descritivas dos dados dos 29 sujeitos buscando evidenciar os resultados gerais nos dois instrumentos. Considerando o caráter diagnóstico da Escala Labirinto, foram realizadas análises estatísticas que compararam os quatro grupos de estudo em relação ao desempenho nas subescalas e na análise total desta escala. Inicialmente, foi realizada a análise estatística descritiva para os dados sociodemográficos e pontuação nos instrumentos utilizados. Para comparar a pontuação nos instrumentos nos diferentes grupos foi realizado o teste não paramétrico de *Kruskal-Wallis*, através do *Jeffreys’s Amazing Statistics Program* (JASP). A identificação das diferenças entre cada grupo utilizado foi calculada por meio do teste *Post Hoc de Connover*.

## RESULTADOS

Na Tabela 1, são expostos os resultados principais nos instrumentos, indicando a condição das crianças em termos do diagnóstico médico/fonoaudiológico, o histórico de sofrimento psíquico e a pontuação obtida na Escala Labirinto em seu total e também nas subescalas.

**Tabela 1:** Descrição dos sujeitos participantes, pontuação no IRDI-Q e na Escala Labirinto, e diagnósticos apresentados

**Sujeito**	**Idade**	**IRDI Q**	**Situação IRDI Q**	**Sintomas centrais Labirinto**					**Labirinto**		**Diagnóstico Profissional**
				**IS**	**CV**		**CNV**	**CRR**	**Total**	**Possui TEA**	
1	2a11m	24	sem sofrimento	12	0	19		13	54	sim	TEA
2	4a9m	35	com sofrimento	0	0	0		0	0	não	AL*
3	4a2m	33	com sofrimento	2	3	2		0	7	não	AL
4	3a6m	39	com sofrimento	0	0	2		0	2	não	DT
5	4a2m	43	com sofrimento	3	1	1		0	5	não	DT
6	4a1m	59	com sofrimento	2	0	0		1	3	não	DT
7	4a6m	35	com sofrimento	0	0	0		0	0	não	DT
8	4a4m	40	com sofrimento	2	3	2		1	8	não	DT
9	4a7m	43	com sofrimento	0	2	0		0	2	não	AL
10	3a5m	30	Sem sofrimento	0	4	5		0	9	não	TDL
11	3a	34	com sofrimento	1	8	5		1	15	não	TDL
12	2a11m	33	com sofrimento	0	3	1		0	4	não	TDL
13	2a11m	19	sem sofrimento	1	7	7		1	16	não	TDL
14	2a11m	60	com sofrimento	3	9	5		3	20	sim	TEA
15	3a3m	46	com sofrimento	11	0	22		11	54	sim	TEA
16	4a9m	40	com sofrimento	3	5	2		3	13	não	TDL
17	3a11m	32	com sofrimento	0	7	1		0	8	não	TDL
18	3a10m	49	com sofrimento	0	0	1		0	1	não	AL*
19	3a10m	44	com sofrimento	0	5	3		0	8	não	TDL
20	3a10m	41	com sofrimento	2	8	6		2	18	não	TDL
21	3a2m	33	com sofrimento	5	9	13		5	32	sim	TEA
22	3ª	71	com sofrimento	9	9	14		9	38	sim	TEA
23	4a4m	57	com sofrimento	5	9	7		5	26	sim	TEA
24	4a11m	18	sem sofrimento	0	9	0		0	9	não	TDL
25	3a4m	27	sem sofrimento	2	9	3		2	16	não	TDL
26	3a6m	56	com sofrimento	4	7	14		4	29	sim	TEA
27	4a6m	52	com sofrimento	1	8	5		1	15	não	AL
28	3a	23	Sem sofrimento	2	5	5		2	14	não	TEA
29	4a	59	Com sofrimento	6	1	11		6	34	sim	TEA

* Teve AL mas não possui mais

Legenda: AL = Atraso de Linguagem; TDL = Transtorno do Desenvolvimento da Linguagem; DT = Desenvolvimento Típico; TEA = Transtorno do Espectro Autista; IS = Interação Social; CV = Comunicação Verbal; CNV = Comunicação Não Verbal; CRR = Comportamento Rígido e Gesto Repetitivo

Na amostra estudada (Tabela 1), dos 29 participantes avaliados, 9 crianças (32%) receberam diagnóstico médico de Transtorno do Espectro Autista (TEA), 10 crianças (36%) foram diagnosticadas com Transtorno do Desenvolvimento da Linguagem (TDL), 5 crianças (18%) apresentaram Desenvolvimento Típico (DT) e 4 crianças (14%) tiveram diagnóstico de Atraso na Aquisição da Linguagem (AL).

Das 9 crianças com diagnóstico médico de TEA, 8 (88,9%) atingiram os critérios diagnósticos na Escala Labirinto. Apenas uma criança (11,1%) não atingiu critério diagnóstico nesta escala no item Interação Social (Sujeito 28). Segundo a terapeuta, esta criança possui TEA nível 1 e recebeu intervenção desde os 11 meses, o que possivelmente explica a pontuação abaixo do ponto de corte.

Na Figura 1, é possível observar a distribuição dos sujeitos, indicando que as crianças diagnosticadas com TEA apresentam pontuações acima do ponto de corte de 12 pontos na Escala Labirinto (8 crianças, 88,9%) e pontuações predominantemente acima de 33 pontos no IRDI-Q (7 crianças, 77,8%). Já entre sujeitos com DT (n=5), AL (n=4) e TDL (n=10), apesar de possuírem histórico de sofrimento psíquico (pontuações entre 33 e 60 no IRDI-Q), o desenvolvimento não indicou psicopatologia grave até o momento da avaliação.

**Figura 1:** Distribuição dos sujeitos nas avaliações a partir do diagnóstico

Legenda: AL = Atraso de Linguagem; DT = Desenvolvimento Típico; TDL = Transtorno do Desenvolvimento da Linguagem; TEA = Transtorno do Espectro do Autismo

Observando as pontuações na Escala Labirinto, as menores médias foram obtidas pelas crianças com DT (Média=3,6; DP=3,0), seguidas pelo grupo AL (Média=5,8; DP=5,8) e pelo grupo TDL (Média=12,7; DP=4,5). As pontuações mais elevadas foram encontradas no grupo TEA (Média=35,4; DP=13,6), como se pode ver na tabela 2.

**Tabela 2:** Médias e desvios padrão dos grupos clínicos na Escala Labirinto

**Grupos Clínicos**	**DT**		**AL**		**TDL**		**TEA**		**Kruskal-Wallis P valor**
	**M**	**DP**	**M**	**DP**	**M**	**DP**	**M**	**DP**	
Interação Social	1,4	1,3	0,6	0,8	0,9	1,1	6,3	3,5	< ,001
Comunicação Verbal	0,8	1,3	2,6	3,2	6,5	2,1	8,7	1,7	< ,001
Comunicação Não Verbal	1,0	1,0	1,6	2,0	3,3	2,3	12,2	5,9	< ,001
Comportamento rígido e repetitivo	0,4	0,5	0,2	0,4	0,9	1,1	6,4	3,7	< ,001
Escala Labirinto	3,6	3,0	5,8	5,8	12,7	4,5	35,4	13,6	

Legenda: DT = Desenvolvimento Típico; AL = Atraso de Linguagem; TDL = Transtorno Desenvolvimento da Linguagem; TEA = Transtorno do Espectro Autista; M = média; DP = desvio padrão

Na Tabela 3, foram realizadas comparações estatísticas entre os grupos clínicos (teste Kruskal-Wallis seguido do teste post-hoc de Conover).

**Tabela 3:** Comparação com teste Post-hoc de Conover, entre os pares de grupos clínicos nas subescalas e na pontuação total da Escala Labirinto

**Escala Labirinto Subescalas e total**	**Comparação dos Grupos**	**z**	**W_i_**	**W_j_**	**p**
Comunicação Verbal	DT – AL	-0,63	480	8,20	0,268
	DT - TDL	-2,48	4,80	16,30	0,262
	DT - TEA	-3,86	4,80	23,00	0,006^*^
	AL - TDL	-1,75	8,20	16,30	< ,001^**^
	AL - TEA	-3,14	8,20	23,00	0,040*
	TDL - TEA	-1,72	16,30	23,00	< ,001**
Interação Social	DT – AL	0,76	13,00	9,00	0,223
	DT - TDL	0,53	13,00	10,55	0,295
	DT - TEA	-2,46	13,00	24,38	0,007*
	AL - TDL	-0,34	9,00	10,55	0,366
	AL - TEA	-3,32	9,00	24,38	< ,001**
	TDL - TEA	-3,6	10,55	24,38	< ,001**
Comunicação Não Verbal	DT – AL	-0,24	7,30	8,60	0,404
	DT - TDL	-1,40	7,30	13,80	0,080
	DT - TEA	-3,57	7,30	24,16	< ,001*
	AL - TDL	-1,12	8,60	13,80	0,131
	AL - TEA	-3,30	8,60	24,16	< ,001**
	TDL - TEA	-2,67	13,80	24,16	0,0004**
Comportamentos restritos e gestos repetitivos	DT – AL	0,32	9,90	8,20	0,371
	DT – TDL	-0,51	9,90	12,20	0,304
	DT - TEA	-3,24	9,90	24,72	< ,001**
	AL - TDL	-0,89	8,20	12,20	0,186
	AL - TEA	-3,62	8,20	24,72	< ,001**
	TDL - TEA	-3,33	12,20	24,72	< ,001**
Pontuação total na Escala Labirinto	DT – AL	-0,42	5,30	7,60	0,335
	DT – TDL	-2,08	5,30	15,00	0,019*
	DT - TEA	-4,04	5,30	24,50	< ,001**
	AL - TDL	-1,58	7,60	15,00	0,056
	AL - TEA	-3,56	7,60	24,50	< ,001**
	TDL - TEA	-2,43	15,00	24,50	0,008*

* p<0,05

** p<0,01

Legenda: DT = Desenvolvimento Típico; AL = Atraso de Linguagem; TDL = Transtorno Desenvolvimento da Linguagem; TEA = Transtorno do Espectro Autista W_i_ e W_j_ soma de postos escore; z = número de desvios padrões acima (positivo) ou abaixo (negativo)

Os resultados na tabela 3 demonstram que, na subescala Comunicação Verbal, houve diferenças significativas entre os grupos DT-TEA (p=0,006), AL-TDL (p<0,001), AL-TEA (p=0,040) e TDL-TEA (p<0,001). Para a subescala Interação Social, destacaram-se diferenças significativas entre DT-TEA (p=0,007), AL-TEA (p<0,001) e TDL-TEA (p<0,001). Na subescala Comunicação Não Verbal, foram encontradas diferenças significativas entre DT-TEA (p<0,001), AL-TEA (p<0,001) e TDL-TEA (p=0,0004). Já na subescala Comportamentos Restritos e Gestos Repetitivos, houve diferenças significativas entre DT-TEA (p<0,001), AL-TEA (p<0,001) e TDL-TEA (p<0,001).

Na análise da Pontuação Total da Escala Labirinto, houve diferenças significativas entre DT-TDL (p=0,019), DT-TEA (p<0,001), AL-TEA (p<0,001) e TDL-TEA (p=0,008).

Esses resultados estão representados visualmente nas Figuras 2 e 3, onde fica evidente o crescimento progressivo da pontuação total da Escala Labirinto desde o grupo com desenvolvimento típico até o grupo com diagnóstico de TEA.

**Figura 2:** Comparação da pontuação total das subescalas

Legenda: CV = Comunicação Verbal; IS = Interação Social; CNV = Comunicação Não Verbal; CRR = Comportamento Rígido e Gesto Repetitivo; DT = Desenvolvimento típico; AL = atraso de linguagem; TDL = transtorno do desenvolvimento da linguagem; TEA = Transtorno do Espectro do Autismo

Na Figura 2, apresenta-se visualmente a comparação entre os quatro grupos clínicos (DT, AL, TDL e TEA) em cada subescala avaliada pela Escala Labirinto (Comunicação Verbal, Interação Social, Comunicação Não Verbal, Comportamento Rígido e Gesto Repetitivo). Observa-se claramente que o grupo com diagnóstico de TEA apresenta pontuações mais elevadas em todas as subescalas comparado aos outros três grupos.

Mais especificamente:

- Comunicação Verbal: Crianças com TEA apresentaram média significativamente superior (Média=8,7; DP=1,7), seguidas por crianças com TDL (Média=6,5; DP=2,1), AL (Média=2,6; DP=3,2) e DT (Média=0,8; DP=1,3).
- Interação Social: Crianças com TEA obtiveram pontuações mais elevadas (Média=6,3; DP=3,5), destacando-se em relação às crianças com TDL (Média=0,9; DP=1,1), DT (Média=1,4; DP=1,3) e AL (Média=0,6; DP=0,8).
- Comunicação Não Verbal: Grupo com TEA mostrou médias significativamente mais altas (Média=12,2; DP=5,9) comparado aos grupos TDL (Média=3,3; DP=2,3), AL (Média=1,6; DP=2,0) e DT (Média=1,0; DP=1,0).
- Comportamento Rígido e Gesto Repetitivo: Novamente, grupo TEA mostrou as maiores pontuações (Média=6,4; DP=3,7), seguido pelos grupos TDL (Média=0,9; DP=1,1), DT (Média=0,4; DP=0,5) e AL (Média=0,2; DP=0,4).

Esses dados reforçam visualmente o potencial discriminativo das subescalas da Escala Labirinto, destacando o perfil diferenciado das crianças com TEA em relação aos demais grupos analisados.

Na Figura 3, observa-se visualmente a comparação entre as pontuações totais dos grupos clínicos (DT, AL, TDL e TEA) obtidas na Escala Labirinto. É possível notar um claro crescimento progressivo nas pontuações totais da Escala Labirinto entre os grupos clínicos.

**Figura 3:** Comparação dos grupos na pontuação total da Escala Labirinto

Legenda: DT = Desenvolvimento típico; AL = atraso de linguagem; TDL = transtorno do desenvolvimento da linguagem; TEA = transtorno do espectro do autismo

Mais especificamente:

- O grupo com Desenvolvimento Típico (DT) obteve a menor pontuação média total (Média=3,6; DP=3,0), seguido pelo grupo com Atraso de Linguagem (AL) (Média=5,8; DP=5,8), pelo grupo com Transtorno do Desenvolvimento da Linguagem (TDL) (Média=12,7; DP=4,5), culminando com o grupo com diagnóstico de TEA, que apresentou a maior pontuação média total (Média=35,4; DP=13,6).

Essa visualização reforça o achado descritivo obtido pela análise estatística (Tabela 3), que mostrou diferenças significativas especialmente claras entre os grupos TEA e os demais grupos clínicos avaliados (DT, AL, TDL).

## DISCUSSÃO

Os resultados obtidos neste estudo demonstraram que a Escala Labirinto^(1)^ conseguiu diferenciar claramente crianças diagnosticadas com Transtorno do Espectro Autista (TEA) das demais condições clínicas avaliadas (TDL, AL e DT). Os achados apontaram diferenças significativas na subescala Comunicação Verbal entre crianças com TEA e as demais, assim como entre os grupos de crianças com Atraso de Linguagem (AL) e Transtorno do Desenvolvimento da Linguagem (TDL). Além disso, as subescalas Interação Social (IS), Comunicação Não Verbal (CNV) e Comportamentos Rígidos e Repetitivos (CRR) destacaram-se pela capacidade de discriminar crianças com TEA dos demais grupos. Observou-se, também, um histórico significativo de sofrimento psíquico no instrumento IRDI-Q^(13)^ para todos os grupos, sendo mais expressivo nas crianças com TEA.

Os resultados encontrados evidenciam que a subescala Comunicação Verbal da Escala Labirinto é sensível na diferenciação clínica entre crianças com TEA e aquelas com TDL, AL e DT. Em particular, notou-se também uma distinção entre crianças com AL e TDL, apontando a subescala como útil na distinção clínica específica entre grupos com queixa de linguagem. Esses achados corroboram os resultados encontrados no estudo realizado por Hage et al.^(6)^, que também identificaram diferenças substanciais nas habilidades pragmáticas entre crianças com TEA, TDL e DT, indicando maior comprometimento pragmático nas crianças com TEA devido às suas dificuldades comunicativas e sociais mais expressivas^(6)^. Os achados deste estudo reforçam a importância clínica da avaliação detalhada das habilidades verbais, contribuindo na identificação precoce e no diagnóstico diferencial do TEA em relação às dificuldades linguísticas isoladas, especialmente em crianças pequenas.

As subescalas **IS, CNV e CRR** revelaram forte potencial discriminativo do grupo com TEA em comparação com os demais grupos clínicos (TDL, AL e DT). Apenas uma criança (sujeito 28) não atingiu critérios diagnósticos, o que pode estar associado à intervenção precoce recebida. Estes achados são consistentes com estudos prévios que validaram instrumentos semelhantes, tais como o ADOS e o ADI-R, que destacam a relevância de déficits sociais, comunicativos e comportamentais para diagnóstico diferencial do TEA^(4,5,12)^. O presente estudo reforça o valor da **Escala Labirinto** enquanto instrumento prático e efetivo no reconhecimento precoce de características típicas do TEA, sendo um recurso acessível, de fácil aplicação e baixo custo, validado especificamente para a realidade clínica brasileira^(1)^.

Este estudo também identificou que crianças com histórico de sofrimento psíquico (avaliado pelo IRDI-Q)^(13)^ podem evoluir sem manifestações clínicas graves, apresentando posteriormente desenvolvimento típico ou com pequenas dificuldades linguísticas. Esses resultados concordam com os achados de Kupfer et al.^(18)^, Nunes et al.^(10)^ e outros estudos subsequentes^(19-21),^ os quais destacam que a presença precoce de sofrimento psíquico nem sempre indica uma trajetória de desenvolvimento patológico. No entanto, a existência desse sofrimento exige atenção clínica especializada, com intervenções precoces e interdisciplinares que possam favorecer desfechos mais favoráveis^(22)^. Assim, esses dados ressaltam a importância da identificação precoce e intervenção integrada com foco na família e na promoção da saúde mental infantil, refletindo diretamente em práticas clínicas preventivas em atenção primária à saúde.

Outro aspecto relevante observado neste estudo refere-se à coexistência de sofrimento psíquico com atrasos e distúrbios de linguagem (AL e TDL). Souza et al.^(9)^ também identificaram relações entre sofrimento psíquico precoce e atrasos de linguagem, sugerindo uma conexão singular entre desenvolvimento psíquico e linguístico. Santos et al.^(23)^ reforçam que a dimensão intersubjetiva influencia significativamente a aquisição da linguagem, ainda que não se estabeleça uma relação direta de causalidade com transtornos do neurodesenvolvimento. Os resultados aqui apresentados reiteram que o desenvolvimento linguístico e psíquico devem ser avaliados conjuntamente, promovendo uma prática clínica interdisciplinar sensível às diferentes trajetórias de desenvolvimento infantil^(22,23)^.

Considerando as limitações deste estudo, especialmente o número reduzido de participantes devido ao contexto pandêmico, são recomendadas futuras pesquisas com amostras maiores, objetivando aprofundar a análise sobre a contribuição diagnóstica diferencial da Escala Labirinto entre TEA e TDL. Além disso, sugere-se estudos populacionais mais amplos sobre aspectos psíquicos e linguísticos na diferenciação entre AL e TDL, auxiliando na compreensão mais refinada dos fatores ambientais e familiares que influenciam a emergência e evolução desses quadros clínicos.

Este estudo contribui para a clínica infantil interdisciplinar brasileira, com implicações diretas na atenção primária e secundária à saúde. Instrumentos como o IRDI-Q^(13)^ mostraram-se eficazes para identificação de histórico de sofrimento psíquico, e a Escala Labirinto^(1)^ no diagnóstico diferencial entre transtornos do neurodesenvolvimento frequentemente confundidos na prática clínica. O fato de a Escala Labirinto ser de fácil acesso a qualquer profissional terapeuta da infância, o seu caráter interdisciplinar, apresentar baixo custo e ser validada para a população brasileira permite afirmar sua potencial utilidade clínica tanto no diagnóstico diferencial quanto no acompanhamento da evolução dos casos clínicos.

Especificamente em relação à linguagem, o item de comunicação verbal da Escala Labirinto se apresentou produtivo tanto na diferenciação entre atraso na aquisição da linguagem e transtorno do desenvolvimento da linguagem, quanto na diferenciação dessas duas condições em relação aos quadros de TEA. A fluidez no diálogo, a presença de linguagem estereotipada e o repertório linguístico considerado a partir de marcos evolutivos de linguagem entre 2 e 4 anos, permitem a diferenciação dos três quadros, pois as crianças com AL e TDL se diferenciam entre si pelos marcos evolutivos, já que as com TDL apresentam alterações mais robustas, com médias muito mais altas nesse item. Também possuem comunicação verbal distinta das crianças com TEA em relação aos três itens, sobretudo quanto ao diálogo e à presença de estereotipias. Sabe-se que quanto mais altas as médias, mais alterada está a subescala.

Outro aspecto potencialmente interessante nos resultados foi a distinção entre as médias de comunicação não verbal, em que as médias das crianças em desenvolvimento típico se assemelham às médias das crianças com atraso na aquisição da linguagem. Já as crianças com TDL apresentaram médias bem mais altas do que esses grupos e bem mais baixas do que as crianças com TEA. Sabe-se que estudos sobre a contribuição da gestualidade na aquisição da linguagem, reforçam a perspectiva multimodal da linguagem^(24)^. Esse resultado demonstra que a comunicação não verbal e verbal integram um quadro maior do funcionamento da linguagem e que as distinções encontradas nessa amostra sinalizam uma potencial distinção entre crianças com AL e TDL no âmbito da gestualidade. Esse resultado coloca em perspectiva pesquisas futuras.

## CONCLUSÃO

Considerando o objetivo inicial desta pesquisa, conclui-se que a Escala Labirinto mostrou-se eficaz na diferenciação entre os grupos investigados (TEA, TDL, AL e DT), tanto em sua pontuação total quanto nas subescalas específicas avaliadas. Os resultados indicam que este instrumento apresenta potencial utilidade clínica, especialmente no diagnóstico diferencial entre o Transtorno do Espectro Autista (TEA) e o Transtorno do Desenvolvimento da Linguagem (TDL). Ademais, a subescala de Comunicação Verbal revelou capacidade específica em distinguir crianças com TEA, TDL e Atraso de Linguagem (AL), sugerindo que sua aplicação complementar a testes específicos de linguagem pode favorecer uma avaliação mais precisa e refinada, auxiliando o fonoaudiólogo na distinção clínica desses quadros na prática clínica.
